# Cell cycle regulation of condensin Smc4

**DOI:** 10.18632/oncotarget.26467

**Published:** 2019-01-08

**Authors:** Hsu Wei-Shan, Vas C. Amit, Duncan J. Clarke

**Affiliations:** ^1^ Department of Genetics, Cell Biology and Development, University of Minnesota Medical School, Minneapolis, MN, USA; ^2^ Present address: Cargill Inc., Wayzata, MN, USA

**Keywords:** condensin, Smc4, Smc2, Mad2, anaphase promoting complex

## Abstract

The condensin complex is a conserved ATPase which promotes the compaction of chromatin during mitosis in eukaryotic cells. Condensin complexes have in addition been reported to contribute to interphase processes including sister chromatid cohesion. It is not understood how condensins specifically become competent to facilitate chromosome condensation in preparation for chromosome segregation in anaphase. Here we describe evidence that core condensin subunits are regulated at the level of protein stability in budding yeast. In particular, Smc2 and Smc4 abundance is cell cycle regulated, peaking at mitosis and falling to low levels in interphase. Smc4 degradation at the end of mitosis is dependent on the Anaphase Promoting Complex/Cyclosome and is mediated by the proteasome. Overproduction of Smc4 results in delayed decondensation, but has a limited ability to promote premature condensation in interphase. Unexpectedly, the Mad2 spindle checkpoint protein is required for mitotic Smc4 degradation. These studies have revealed the novel finding that condensin protein levels are cell cycle regulated and have identified the factors necessary for Smc4 proteolysis.

## INTRODUCTION

An essential cell cycle event for maintaining genome stability is chromosome condensation, a process in which the interphase chromatin condenses into highly compacted chromosome during mitosis. In most organisms, the interphase chromatin begins to compact in prophase and becomes visible as bar-shaped mitotic chromosomes in metaphase. Condensin is a key factor in the condensation process and is one of the most abundant chromosome associated proteins [1-3]. In human cells, there are two types of condensin complex, type I and type II [4]. Type II condensin (condensin II) constitutively exists in the nucleus, whereas condensin I locates in the cytoplasm until nuclear envelope break down when condensin I is able to access chromosomes to maintain and strengthen the condensed state [5]. Only homologues of condensin I are found in *Saccharomyces cerevisiae* [6]. However, unlike mammalian condensin I, the condensin complex in budding yeast is known to be in the nucleus throughout the cell cycle [7]. Therefore, it is clear that the physical shield of the nuclear envelope is not the mechanism which regulates condensin activity, such that chromosome condensation is limited to mitosis in budding yeast.

Budding yeast condensin is composed of an Smc2-Smc4 heterodimer and three non-Smc subunits, Brn1, Ycs4 and Ycg1 [7-10]. Except for *BRN1*, the transcripts of condensin genes only slightly oscillate through the cell cycle [11]. Indeed, studies of the regulation of condensin in other species have largely focused on post-translational mechanisms [6]. It is well established that mitotic Cyclin dependent kinases (Cdks) promote chromosome condensation from yeast to human [12-15]. However, the substrate(s) of Cdks important for chromosome condensation are unknown. *In vitro*, purified mitotic-specific Cdk (Cdk1-cyclin B) phosphorylates three non-Smc subunits of Xenopus and human condensin [14, 16]. In addition, several proteome-wide studies identified two phosphorylated sites within Cdk consensus sequences of the human Smc4 protein, suggesting that human Smc4 might be a target for Cdk. Only Smc4 and one non-Smc subunit in fission yeast contain Cdk consensus sites [13]. Cdc2-dependent phosphorylation of T19 on Cut3/Smc4 in fission yeast has been shown to induce the translocation of the condensin complex from cytoplasm to nucleus to promote chromosome condensation [13]. In budding yeast, Cdk consensus sequences are present in four condensin subunits, although Smc4 is the only condensin subunit that was found to be a Cdk substrate in a proteome wide screen [17]. Two further phosphoproteome studies revealed possible Cdk phosphorylation sites in budding yeast Smc4 [18, 19]. The identified phosphorylation sites within a Cdk consensus sequence in Smc4 among these different species are always clustered at the non-conserved N-terminal region. These findings suggest that Smc4 might be the conserved condensin subunit regulated by Cdk.

In this study, we mutated the putative phosphorylation sites of Smc4 and found that they are dispensable for condensation, and unexpectedly the phosphor-mutant allele encoded a form of Smc4 that was more stable than the wild type protein. Strikingly, Smc4 protein levels oscillated through the cell cycle, peaking at the time of mitosis when chromosomes condensed. Decondensation was accompanied by APC/C- and Mad2-dependent degradation of Smc4. In summary, we made the unexpected observation that Smc4 is regulated at the level of protein stability, in part regulated by Cdk phosphorylation, and in mitosis by APC/C and Mad2.

## RESULTS

### Smc4 CDK sites are dispensable for chromosome condensation

**Figure 1:**
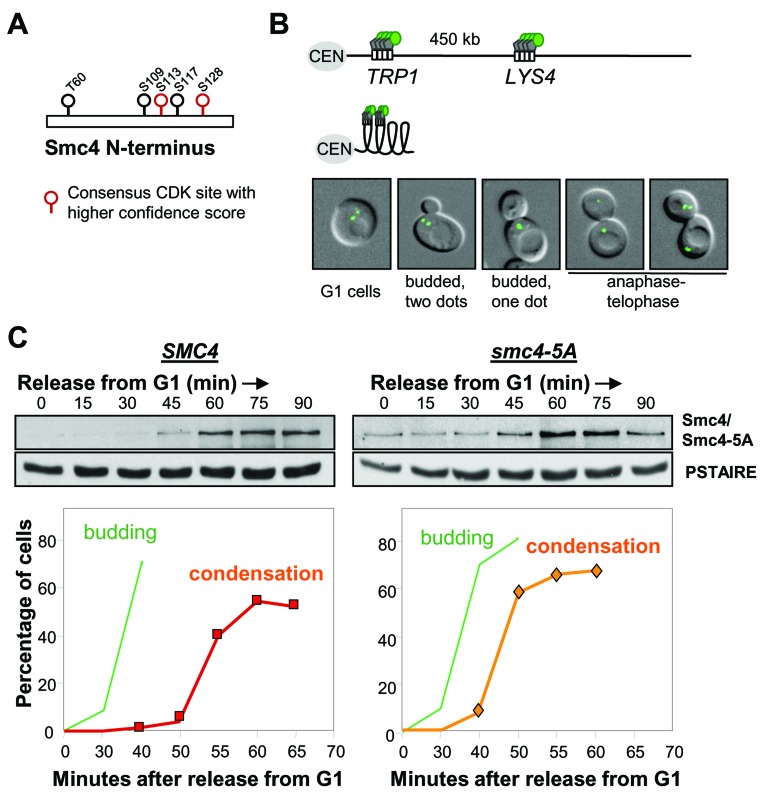
Smc4 CDK sites are dispensable for chromosome condensation **A.** Cdk full consensus sequences in S. cerevisiae Smc4. Solid circles indicate residues known to be phosphorylated; determined by proteome-wide analysis (see text). Residues with higher confidence scores are shown in red. **B.** Cartoon showing the LacO/GFP-LacI system used for the condensation assay. Two-separated GFP signals can be detected on uncondensed right arm of chromosome IV (Top). Condensed chromosome IV brings two GFP signals together (Bottom). White rectangle indicates Lac operator sequence. Gray pentagon indicates Lac repressor protein. Green circle indicates green fluorescence protein. CEN: centromere. The images are wild type yeast cells with GFP marked *TRP1* and *LYS4* loci in various stages of the cell cycle. From left to right: G1 (unbudded with two GFP dots), S (Small bud with 2 GFP dots), G2/M (budded with one GFP dot, indicating chromosome condensation) and Anaphase/Telophase (one or 2 GFP dots in each daughter cell). **C.** Analysis of a synchronous cell cycle after G1 arrest (mating pheromone) in wild type and *smc4-5A* cells. After releasing from G1 arrest, samples were taken for scoring budding (green) and chromosome condensation (red/orange). The Western blots show wild type Smc4 and Smc4-5A protein levels. PSTAIRE is the loading control.

In *S. cerevisiae*, the Cyclin dependent kinase (Cdk), Cdc28, is required for the linear compaction of chromosomes that occurs shortly before anaphase [15]. The core condensin subunit, Smc4, has five full Cdk consensus sites (S/T-P-X-R/K) which are clustered in the non-conserved N-terminal region (Figure 1A). Four of these sites were identified as *bona fide* Cdc28 substrates in a proteome-wide study [18]. In order to understand the function of Cdk-dependent phosphorylation of Smc4, we mutated all five full Cdk consensus residues to mimic the lack of Cdk phosphorylation by replacing the corresponding serine or threonine residues with alanine (*smc4-5A*). The Smc4 N-terminus carrying these five alanine substitutions was then integrated to replace the endogenous *SMC4* locus and generate the *smc4-5A* allele expressed from the native promoter. Strains harboring this allele were viable and were not temperature sensitive (data not shown), indicating that these five phosphorylation sites are dispensable, whereas *smc4* null cells are inviable [20]. We then monitored mitotic chromosome condensation in *smc4-5A* mutant cells using an assay previously developed in which the coalescence of loci on the long arm of Chr. IV can be directly visualized in live cells [15] (Figure 1B). Cells were released from G1 synchrony, following mating-pheromone induced arrest, then Smc4 protein levels were monitored by Western blotting and the timing of condensation was determined by live cell microscopy (Figure 1C). In wild type, chromosome condensation, as indicated by the emergence of budded cells with a single GFP dot, was first observed 55 minutes after release from G1. This matched an increase in the protein level of Smc4, suggesting that the abundance of Smc4 might be one mechanism which controls the onset of chromosome condensation. Consistent with the viability of *smc4-5A* cells, Chr. IV condensed similar to wild type cells. In fact relative to the timing of bud emergence, condensation was marginally premature in *smc4-5A* mutant cells (Figure 1C). This premature condensation phenotype was reproducible in three independently isolated *smc4-5A* strains, but was not observed in a control strain in which the wild type N-terminus of *SMC4* was integrated into the genome using the same strategy as for the *smc4-5A* mutant (data not shown). Considering that chromosomes fail to condense in temperature sensitive *cdc28* mutants [15], the Smc4 Cdk sites cannot be the Cdc28 targets for initiating condensation. The data do indicate, however, that these residues affect the timing of chromosome condensation, though this is not important for cell viability.

### Smc4 protein abundance is cell cycle regulated

**Figure 2:**
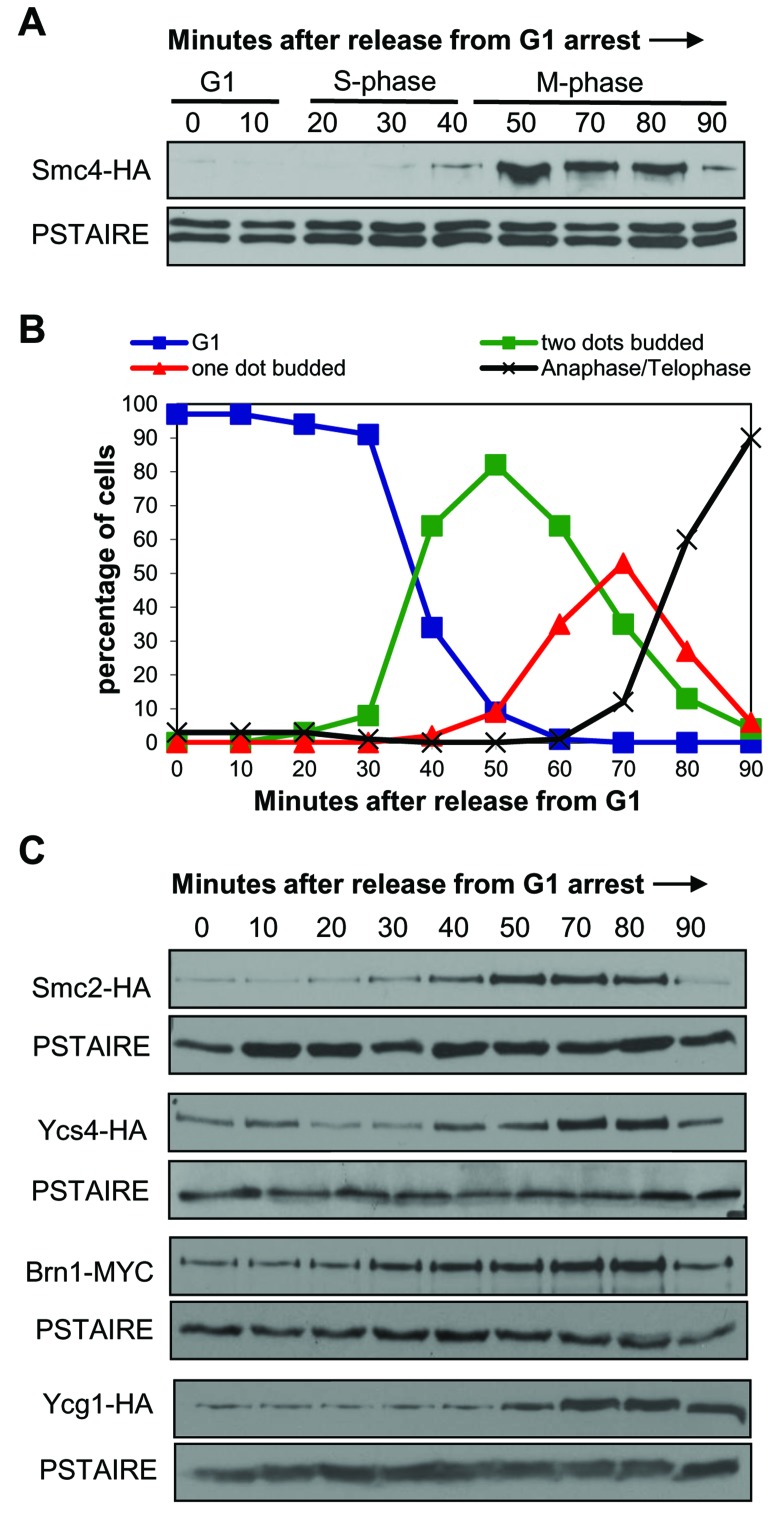
Protein levels of condensin subunits are cell cycle regulated **A.** Western blot analysis of Smc4 levels in a synchronous cell cycle. Smc4-HA cells were synchronized in G1 with alpha-mating pheromone then samples taken every 10 minutes after release from G1. PSTAIRE recognizes Cdc28 protein as the loading control. The cell cycle stage labels show the approximate cell cycle timing based on the known relationship between budding, DNA replication and mitosis. **B.** Timing of condensation in the Smc4-HA strain in a synchronous cell cycle experiment as in part A. After releasing from G1, samples were taken for scoring the cell morphology and condensation as described in Figure 1. **C.** Western blot analysis of Smc2-HA, Ycs4-HA and Brn1-Myc levels in a synchronous cell cycle as described in part A.

The analysis of Smc4 and Smc4-5A protein abundance using synchronized populations revealed an oscillatory pattern through the cell cycle with the peak protein level coinciding with the observation of condensed chromosomes (Figure 1). This suggests that the abundance of Smc4 might be one mechanism which controls the onset of chromosome condensation. The Smc4-5A protein levels were slightly higher in G1 and S-phase cells (0-45 min) compared to the corresponding wild type populations, perhaps contributing to the slightly premature chromosome condensation. Because this oscillatory pattern had not been previously described, we performed more detailed time course experiments and also examined the abundance of other condensin subunits though the cell cycle (Figure 2). The Smc4 protein level was low in G1 and S-phase cells, peaking between 50-80 minutes following release from G1 arrest (Figure 2A). The peak abundance coincided with condensation (Figure 2B). We also observed a decrease in the Smc4 protein level at a time (90 min) when most cells had progressed into anaphase. A similar oscillation was observed for the other SMC condensin subunit Smc2, while the non-SMC subunits Ycs4, Ycg1 and Brn1 oscillated to a lesser degree (Figure 2C).

**Figure 3:**
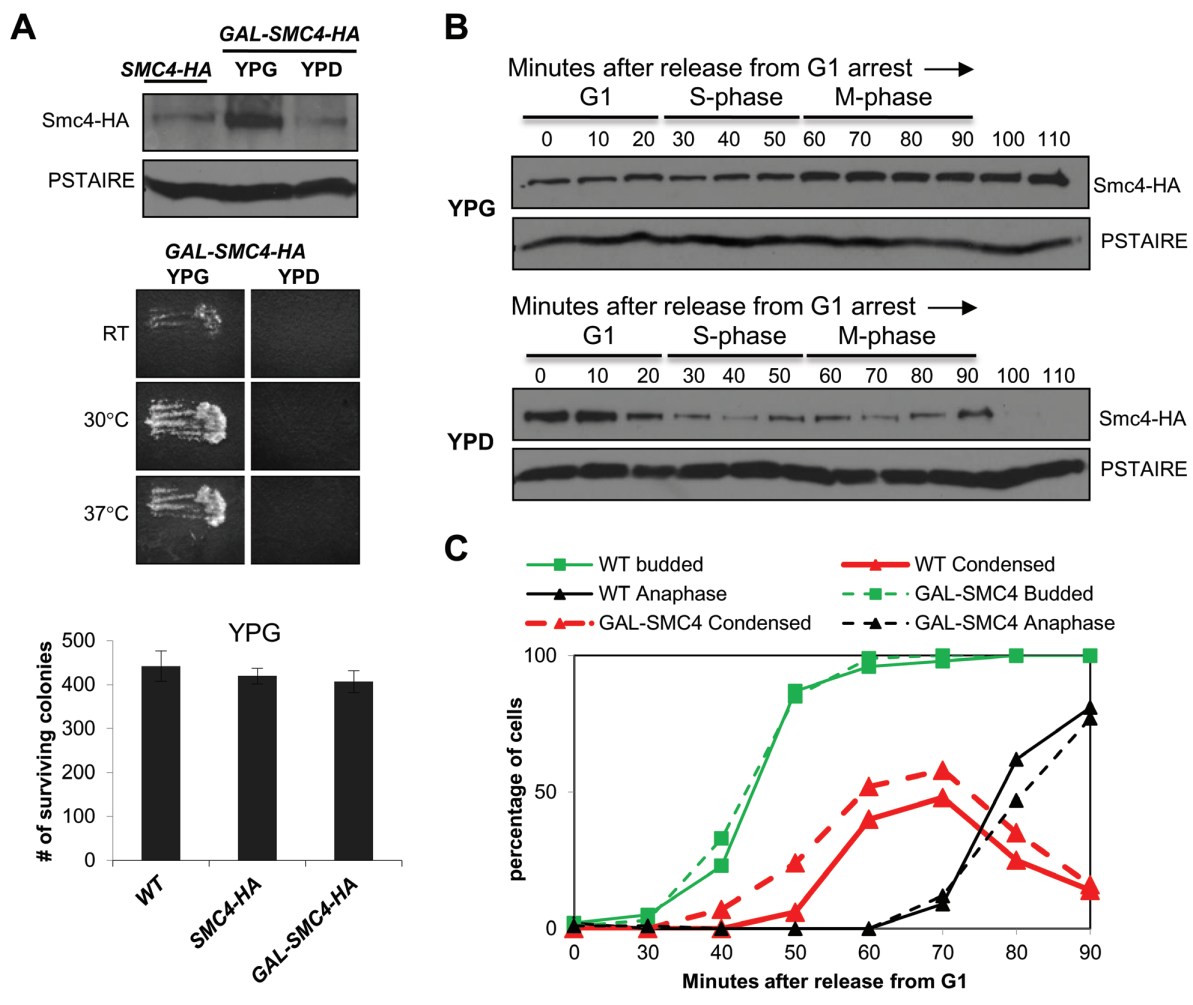
Smc4 protein is degraded in mitosis **A.** Upper panel: Western blot analysis of Smc4 protein level in *SMC4-HA* and *GAL-SMC4-HA* strains. Overnight cultures were grown in medium containing galactose (YPG medium). After diluting, the *SMC4-HA* culture was maintained in medium with galactose for 3 hours, whereas the *GAL-SMC4-HA* culture was grown in medium containing galactose (YPG) or dextrose (YPD). PSTAIRE recognizes Cdc28 protein as the loading control. Middle panel: Replica patching assay indicating *GAL-SMC4-HA* viability on rich medium with galactose (left) and lethality on dextrose (right) at RT, 30°C, 37°C. Bottom panel: Colony survival of wild type, *SMC4-HA* and *GAL-SMC4-HA* strains plated on galactose containing rich medium. Cells in log phase (1 OD) in rich liquid (YPG) medium were counted and 500 cells were plated in six replicates. Number of surviving colonies was determined after 5 days at 30°C. **B.** Analysis of a synchronized cell cycle after G1 arrest of the *GAL-SMC4-HA* strain and release into galactose or dextrose containing rich medium. After release, samples were taken every 10 minutes and subjected to Western blot analysis for Smc4 protein level. PSTAIRE recognizes Cdc28 protein as the loading control. The cell cycle stage labels show the approximate cell cycle timing based on the known relationship between budding, DNA replication and mitosis. **C.** Analysis of a synchronous cell cycle after G1 arrest in rich (YPG) medium in wild type and *GAL-SMC4-HA* strains. After releasing from G1 arrest, samples were taken for scoring budding (green), chromosome condensation (red) and anaphase (black) as described in Figure 1.

Global analysis of the transcriptome in budding yeast revealed that condensin gene expression is relatively uniform through the cell cycle [11]. Presumably, the oscillation in Smc2 and Smc4 is due to regulation at the level of protein translation or stability. To confirm this we examined Smc4 protein levels in promoter shut-off experiments. We constructed a galactose inducible HA tagged allele of Smc4, in which the endogenous *SMC4* promoter was replaced by the *GAL1* promoter. In asynchronously growing cells, Smc4 was more abundant when expressed from *GAL1* than from the endogenous promoter but the level was quickly reduced in the presence of dextrose which silences *GAL1* (Figure 3A). The *GAL-SMC4-HA* strain was viable and was not temperature sensitive when grown with galactose, but was inviable when dextrose was the carbon source (Figure 3A). Therefore, cells tolerate excess Smc4. To determine the contribution of protein translation and/or stability to Smc4 cell cycle oscillation, we released *GAL-SMC4-HA* cells from G1 arrest into medium containing galactose (YPG) or dextrose (YPD) (Figure 3B). In YPG the abundance of Smc4 remained constant from G1 to S-phase, but increased slightly in M-phase, consistent with the observation that Smc4 protein peaked in mitosis when expressed from the endogenous promoter. In contrast, when *GAL1* was repressed upon G1 release, Smc4 protein abundance fell in S-phase and was undetectable by the time the cells reached late M-phase (100-110 min). These data are consistent with reduced Smc4 stability in S-phase and degradation of the remaining Smc4 protein in late mitosis.

We also monitored chromosome condensation in the *GAL-SMC4-HA* strain to ask if excess Smc4 protein affects the timing of chromosome condensation. The *GAL-SMC4-HA* strain was subjected to the condensation assay as described in Figure 1, except that the cells were grown in YPG medium. We observed that chromosome condensation was slightly premature, similar to the phenotype seen in *smc4-5A* cells (Figure 3C). Each of these results indicates that elevated levels of Smc4 can drive early condensation, but that cell viability is largely not affected.

### Chromosome decondensation correlates with Smc4 degradation

**Figure 4:**
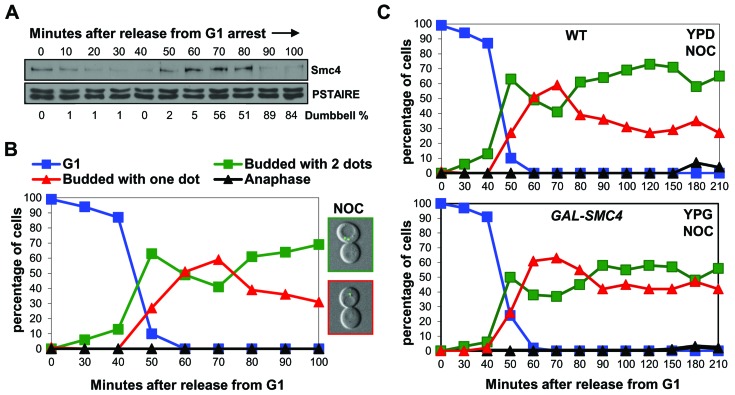
When chromosomes decondense in nocodazole arrested cells, Smc4 protein is degraded **A. and B.** Analysis of a synchronous cell cycle after G1 arrest in rich (YPD) medium with nocodazole treatment in wild type cells. Nocodazole was added 10 minutes after releasing from G1 arrest. Samples were taken for Western blot for Smc4 protein level **A.,** and for scoring condensation **B.,** as described in Figure 1. The microscopy images on the right in **B.,** indicate the pre-anaphase arrested cells with decondensed (Top) or condensed chromosomes (Bottom). **C.** Analysis of a synchronous cell cycle after G1 arrest of wild type and *GAL-SMC4* strains in rich medium with nocodazole treatment. Nocodazole was added 10 minutes after releasing from G1 arrest. Samples were taken at indicated times after release for scoring condensation as described in Figure 1.

The above studies indicated that Smc4 is degraded in mitosis around the time of mitotic exit when chromosomes decondense. Previous studies showed that, unlike in most eukaryotic cells, budding yeast cells that become arrested in mitosis before anaphase fail to maintain chromosome condensation [15]. We asked if the abundance of condensin Smc4 correlates with the status of chromosome condensation upon mitotic arrest. Cells were released from G1 synchrony in the presence of nocodazole to induce mitotic arrest. Chromosome condensation was observed after 50-70 minutes, similar to in the absence of nocodazole, and the fraction of the budded cells with condensed chromosomes decreased from 80 minutes onwards (Figure 4A, 4B). Interestingly, decondensation approximately coincided with a decrease in the abundance of Smc4 protein (Figure 4A, 4B). Moreover, when the experiment was repeated in the *GAL-SMC4-HA* strain, decondensation was partially delayed in the presence of nocodazole (Figure 4C). These experiments indicate that the failure of budding yeast cells to maintain condensation during mitotic arrest is in part due to degradation of Smc4. This conclusion is in agreement with other work showing that the maintenance of condensation during an unperturbed mitosis requires active condensin complexes [21, 22].

### Smc4 degradation in mitosis requires proteasome activity

**Figure 5:**
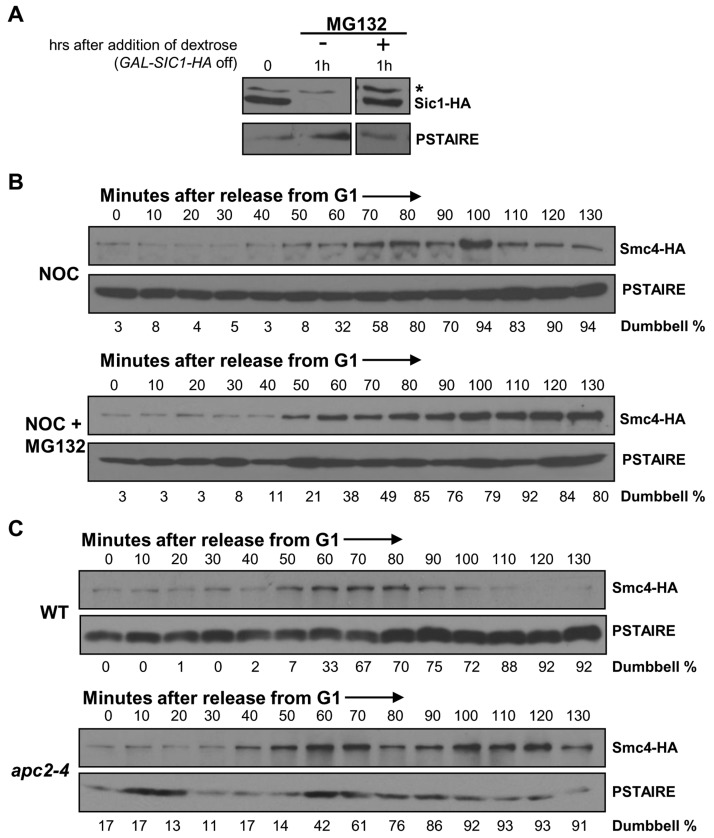
Smc4 protein degradation requires APC/C ubiquitin ligase **A.** Analysis of Sic1-HA protein stability to confirm the efficiency of proteasome inhibition by MG132 in a *rpn4∆ pdr5∆* strain. Cells harboring a *GAL-SIC1-HA* plasmid were cultured in galactose containing rich medium to induce Sic1-HA protein production for 45 minutes, followed by switching to dextrose containing rich medium to turn off Sic1-HA production and adding MG132 to half of the culture to inhibit proteasome activity. Samples were collected an hour after the addition of MG132 and subjected to Western blot analysis for detecting Sic1-HA. The asterisk indicates a non-specific band. PSTAIRE, loading control. **B.** Analysis of a synchronized cell cycle after G1 arrest in rich medium in *rpn4∆pdr5∆ SMC4-HA* cells with or without proteasome inhibitor MG132. After G1 arrest with mating pheromone, cells were released into rich medium with the addition of nocodazole 10 minutes after the release. MG132 was added to half of the culture to inactive proteasome activity. Samples were taken every 10 minutes and subjected to Western blot analysis for Smc4 protein level. PSTAIRE, loading control. **C.** Analysis of a synchronized cell cycle after G1 arrest in rich medium in *SMC4-HA* and *apc2-4 SMC4-HA* strains. Cells were arrested in G1 with mating pheromone at the permissive temperature for 2 hours followed by an additional half hour at the non-permissive temperature to inactive Apc2 function. Cells were then released into cell cycle at the non-permissive temperature with the addition of nocodazole to both strains at 10 minutes after the release. Samples were taken every 10 minutes after the release and subjected to Western blot analysis for Smc4 protein level. PSTAIRE, loading control.

The data presented above indicate that Smc4 protein abundance regulates the status of chromosome condensation. The significant decline of Smc4 protein at the end of mitosis or during pre-anaphase arrest implies a requirement for rapid protein degradation to revert condensation at the end of mitosis. We therefore asked if the proteasome is required for the drop in Smc4 abundance. For these experiments, strains with the *rpn4Δ pdr5Δ* combination were utilized and the proteasome was inhibited with MG132 during pre-anaphase arrest. *Rpn4* is a transcription factor that up-regulates genes encoding multiple proteasome subunits [23]. Pdr5 is a multi-drug transporter [24]. Loss of both of these factors allows inhibition of proteasome activity by MG132, though even in these strains proteasome activity is not completely abolished [23, 25]. As a control we first examined the stability of Sic1, a well characterized protein that is degraded by the proteasome [26]. In strains harboring a galactose inducible *SIC1-HA* gene, proteasome dependent degradation was observed within 1 hour of promoter shut-off in the presence of dextrose (Figure 5A).

We then released *SMC4-HA rpn4Δ pdr5Δ* cells from G1 synchrony in the presence of nocodazole to arrest cells in the subsequent mitosis, either in the presence of absence of MG132. Nocodazole was used to arrest the cells in mitosis under each condition and thus eliminate possible cell cycle position effects. As predicted, due to reduced proteasome activity in the *rpn4∆ pdr5∆* cells, Smc4 levels were elevated even in the absence of MG132 (Figure 5B). Moreover, the presence of MG132 further stabilized Smc4 protein during the pre-anaphase arrest (Figure 5B). These data indicate a requirement of proteasome activity for Smc4 protein turn over in mitosis.

### Smc4 degradation requires APC/C ubiquitin ligase

The timing of Smc4 degradation, which occurs around the time of anaphase in an unperturbed cell cycle, suggests a requirement for the APC/C ubiquitin ligase. A temperature sensitive mutant of a catalytic core subunit of the APC/C ubiquitin ligase, *apc2-4* [27], which inactivates both Cdc20 and Cdh1 dependent APC/C ubiquitin ligase activity at the non-permissive temperature was used to test this prediction. The Smc4 protein level was monitored after release from G1 arrest in wild type and the *apc2-4* mutant at the non-permissive temperature and in the presence of nocodazole to eliminate cell cycle position effects (Figure 5C). Both wild type and *apc2-4* cells entered S-phase after ∼30 minutes (data not shown). Accumulation of dumbbell shaped cells was equivalent, indicating similar cell cycle progression in each strain. As expected, the Smc4 protein level during pre-anaphase arrest in wild type cells peaked during the interval from 50 to 80 minutes, followed by degradation of Smc4. However, the Smc4 protein remained at a high level in the *apc2-4* cells at least until 130 minutes after G1 release. Therefore APC/C-dependent ubiquitin ligase activity is likely required for Smc4 protein turnover.

### Smc4 degradation requires Mad2

**Figure 6:**
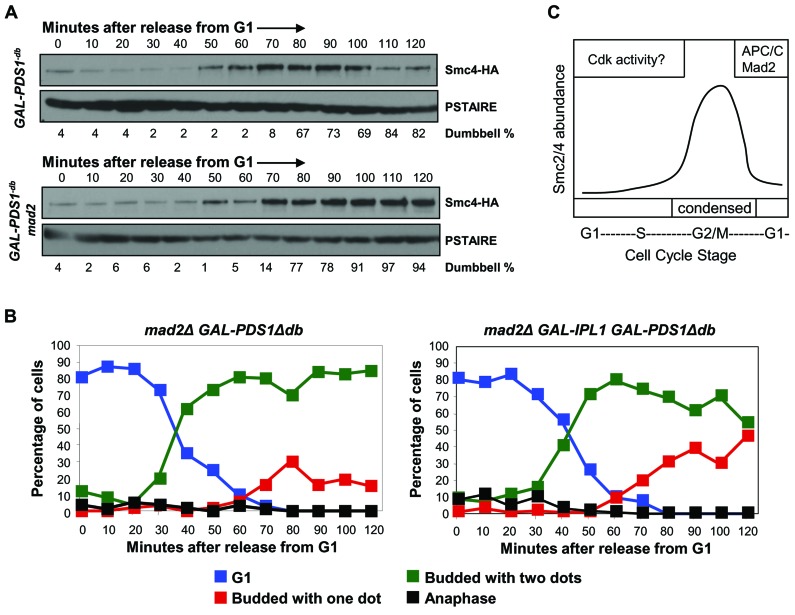
Mad2 is required for Smc4 degradation in mitosis **A.** Analysis of a synchronized cell cycle after G1 arrest in rich medium in *SMC4-HA GAL1-PDS1Δdb* and *mad2Δ SMC4-HA GAL1-PDS1Δdb* strains. Cells were arrested in G1 with mating pheromone in raffinose rich medium. Galactose was added 15 minutes after the release to induce expression of the destruction box mutant form of Pds1. Cells were collected and then subjected to Western blot analysis for detecting Smc4 protein level. **B.** Analysis of a synchronized cell cycle after G1 arrest in rich medium in *mad2∆ GAL-PDS1Δdb* and *mad2Δ GAL-PDS1Δdb GAL-IPL1* strains. Cells were arrested in G1 with mating pheromone in raffinose rich medium. Galactose was added 15 minutes after the release to induce expression of the destruction box mutant form of Pds1 and Ipl1 kinase. Samples were taken at indicated times after release for scoring condensation as described in Figure 1. **C.** Proposed model to explain the cell cycle regulation of Smc4 protein. Phosphorylation of CDK sites in the N-terminal region lead to partial destabilization Smc4 in G1 and S-phase. In mitosis, Smc4 is degraded to undetectable levels dependent on the APC/C and Mad2.

The requirement of APC/C ubiquitin ligase for Smc4 protein turnover at the end of the cell cycle matches the active window of APC/C ubiquitin ligase. The APC/C ubiquitin ligase is active from the onset of anaphase to the next G1-S transition, but is activated sequentially through two substrate specificity factors, Cdc20 and Cdh1 [28, 29]. Cdc20, but not Cdh1, is inhibited by Mad2, which is the effector molecule of the spindle assembly checkpoint. We predicted that Mad2 would function to stabilize Smc4 to allow chromosomes to condense, and remain so, under conditions where cells are arrested before anaphase by the spindle checkpoint. In order to test this we asked if Smc4 protein is degraded prematurely in the absence of Mad2 protein. We compared the Smc4 protein level in a wild type and a *mad2Δ* strain after release from G1 synchrony. A galactose inducible destruction box mutant form of Pds1 [30] was used to achieve a pre-anaphase arrest, in order to eliminate cell cycle position differences between these two strains. The cells were arrested in G1 in raffinose rich medium, then galactose was added 15 minutes after the release to induce the production of non-degradable Pds1 protein. Surprisingly, instead of earlier degradation, we found that Smc4 was stabilized in the absence of Mad2 (Figure 6A). Under these conditions, Cdc20-dependent APC/C ubiquitin ligase should be active due to the lack of Cdc20 sequestration by Mad2. Given that the APC/C ubiquitin ligase is responsible for Smc4 degradation (Figure 5), it is unclear why Smc4 was stabilized when Cdc20-dependent APC/C ubiquitin ligase is robustly active. Nevertheless, this result suggests that Mad2 promotes Smc4 degradation at the end of the cell cycle instead of preventing Smc4 from proteolysis before anaphase.

Since Smc4 was stabilized in the absence of Mad2 we asked if chromosome condensation was maintained when *mad2∆* cells are arrested in the presence of non-degradable Pds1 protein. However, under these conditions condensation was not maintained (Figure 6B). Therefore, similar to the data described above, stabilization of Smc4 alone is not sufficient for robust maintenance of condensation (Figure 4C). Previous studies have shown that the Aurora B kinase (Ipl1 in budding yeast) functions to maintain condensation in anaphase cells [15, 31]. We therefore asked if condensation is maintained in *mad2∆* cells, arrested in the presence of non-degradable Pds1 protein, when the Ipl1 kinase is over-produced. Indeed, under the same growth conditions, cells expressing *IPL1* from the *GAL1* promoter maintained condensation to a greater extent (Figure 6B). Therefore, Ipl1 kinase and Smc4 stability are determinants of the condensation status during mitotic arrest in budding yeast.

## DISCUSSION

Although it is known that Cdk initiates chromosome condensation in eukaryotes [6], the condensin substrates regulated by Cdks remain ambiguous. In budding yeast, Smc4 is the only condensin subunit identified as a Cdk substrate [17]. However, unlike *cdk* mutants, *smc4-5A* which bears alanine substitutions of the five Cdk consensus residues (within full consensus sequences) was fully competent to condense chromosomes in mitosis. Therefore, the essential Cdk site(s) in Smc4 for initiating chromosome condensation are not any of these five full Cdk consensus sites. The possibility remains that minimal Cdk consensus sites in Smc4 are required for chromosome condensation. Alternatively, other condensin subunits might be the Cdk target in budding yeast, although all of the other subunits have few Cdk consensus sequences. A study of human condensin II provided evidence that phosphorylation of Cap-D3, one of the HEAT motif containing non-Smc subunits, promotes early stages of chromosome condensation in prophase [32]. Although this subunit in human condensin II is not conserved in budding yeast, it is still possible that the counterpart component in budding yeast, Ycs4, which contains one full and four minimal Cdk consensus sequences, is subjected to Cdk regulation for inducing chromosome condensation. However, even in the case of Cap-D3 phosphorylation by Cdk, condensation was only partially affected. It is therefore likely that Cdk activates condensin through multiple targets, some of which may indirectly affect condensin complex activity.

Although Smc4-5A did not assemble into complexes that are defective for performing condensation, the protein was more stable than WT Smc4. This was evident in early S-phase of the cell cycle when Smc4 has reduced stability. This finding suggests that the phosphorylation of Smc4 on these five Cdk sites plays a role in Smc4 degradation. Consistent with the increased stability in S-phase, *smc4-5A* cells, as well as cells over-producing Smc4, had a mild phenotype in which condensation was premature. This early condensation was reproducible but occurred only about 5 minutes before condensation in wild type cells relative to bud emergence. Presumably the increased abundance of Smc4, just before mitosis, can influence the timing of condensation but is not the sole requirement for condensation. Most likely, the other condensin subunits, which also oscillate to various extents, must reach threshold levels. Indeed, a recent study of the yeast Ycg1 subunit of condensin provided compelling evidence that it is regulated both at the transcriptional level and at the level of protein degradation, impacting condensin complex function [33]. The same study did not observe oscillation of Smc4 protein levels through the cell cycle. This indicates that different condensin subunits may potentially be regulated among genetically diverse yeast strain background. It is also possible that budding yeast rely more heavily on oscillating condensin abundance to regulate condensation than do other eukaryotes. Unlike most eukaryotic cells, budding yeast B-type cycle/Cdk is active before mitosis. This could explain why Ycg1 and Smc4 condensin abundance can be regulated in interphase to prevent premature condensation.

In mitosis, Smc4-5A was degraded following its peak in abundance around the time of metaphase/anaphase. Therefore, the putative phosphorylation-dependent degradation of Smc4 that contributes to the low levels in S-phase and G1 is likely to be irrelevant in mitosis when degradation is due to a distinct mechanism. Examination of wild type Smc4 protein abundance in synchronized cell cycle experiments revealed rapid degradation in mitosis. Since transcript levels are relatively flat through the cell cycle [11], Smc4 proteolysis induced by the APC/C and proteasome is likely the major mechanism that allows decondensation after anaphase. The proteolysis of Smc4 correlated with the timing of decondensation after anaphase and also occurred under mitotic arrest conditions where, unusually, the chromosomes of budding yeast undergo decondensation. We did not, however, detect accumulation of Smc4 protein in asynchronous cultures of either a temperature sensitive *cdc20-1* mutant or a *cdh1Δ* mutant (data not shown). This suggests the involvement of both Cdc20 and Cdh1-dependent APC/C ubiquitin ligase in Smc4 degradation. The Cdh1-dependent APC/C ubiquitin ligase might take over the function of Cdc20-APC/C at the end of anaphase and into G1 phase, when Cdc20 is targeted for degradation by Cdh1-APC/C [34, 35]. In support of this, a D box motif, which can be recognized by Cdc20 or Cdh1, and a KEN box motif, a Cdh1 specific recognition sequence [36], are found in Smc4.

A striking observation was that Mad2 was required for Smc4 degradation. In the case of most APC/C substrates, the Mad2 spindle checkpoint protein is associated with Cdc20 inhibition, and thus stabilization of APC/C substrates. However, with similarity to the mechanism of Smc4 degradation, Cdc20 protein degradation has been reported to be induced by the APC/C through a Mad2-dependent mechanism [37]. When the spindle checkpoint is active, the phosphorylation of Cdc20 by p38 MAPK promotes the formation of Cdc20-Mad2-APC/C complexes which are a prerequisite for Cdc20 degradation [38]. Perhaps through a similar process, Smc4 proteolysis requires Mad2. In this scenario, one prediction is that Smc4 interacts with the APC/C in a manner that is enhanced by active Mad2.

During mitotic arrest in the presence of stable Pds1, we observed that condensation is not maintained even when Smc4 levels remained high (in *mad2∆* cells). Consistent with other studies however [15, 31], over-production of Ipl1 kinase delayed decondensation under these conditions (Figure 6B). Based on these data it is clear that multiple factors combine to preserve condensation, including the mechanisms described here that stabilize the condensin subunits, as well as Ipl1 and Cdk kinase activities.

In summary, we have presented evidence that the protein level of the condensin subunit Smc4 is cell cycle regulated, peaking at the time of condensation in mitosis (Figure 6C). The decline of Smc4 protein following anaphase is consistent with APC/C ubiquitin ligase mediated proteolysis. Unexpectedly, the data also indicate a positive role of Mad2 in the mechanism of Smc4 degradation. In addition to Cdc20, Smc4 may therefore be another example of an APC/C substrate that requires Mad2 for its proteolysis.

## MATERIALS AND METHODS

### Yeast strains

Strains were derived from BF264-15 15DU: *MATa ura3Δns ade1 his2 leu2-3, 112 trp1-1a* [39] and were grown at 30°C in medium containing yeast extract and peptone plus dextrose (YPD), galactose (YPG) or raffinose (YPR). To generate the *GAL1-SMC4* allele, an integrating DNA fragment containing *KAN*^*R*^ and the *GAL1* promoter flanked with sequences with homology to *SMC4* promoter region and start codon were amplified from pFA6a-kanMX6-PGAL1 [40] using the following primer pairs: SMC4-5’-F4: 5’TCTACACACTGGAATCGA TTTCATACCATAAAAGAGGCCCgaattcgagctcgtttaaac 3’, SMC4-3’-R2: 5’CGGACTTCCGCTTTTGT CTTTTGCTCAATGGACTATCAGAcattttgagatccgggtttt 3’ (Sequence homology to *SMC4* is shown in caps, sequence homology to *KAN*^*R*^ gene or *GAL1* promoter shown in lowercase) (Table 1).

**Table 1:** Strain Genotypes

Strain	Genotype
4128	*MATa bar1Δ SMC4-6HA::TRP1 trp1::LacO(TRP1, LEU2) lys4::LacO(LEU2) his3::LacR- GFP(HIS3)*
	*MATa bar1Δ smc4-5A-6HA::TRP1 trp1::LacO(TRP1, LEU2) lys4::LacO(LEU2) his3::LacR- GFP(HIS3)*
AVY211	*MATa bar1Δ SMC2-6HA::TRP1 ura3::HIS3:GFP-TUB1(URA3)*
AVY222	*MATa bar1Δ BRN1-13MYC ura3::HIS3:GFP-TUB1(URA3)*
AVY220	*MATa bar1Δ YCS4-HA ura3::HIS3:GFP-TUB1(URA3)*
4294	*MATa bar1Δ GAL-SMC4-6HA::TRP1 trp1::LacO(TRP1, LEU2) lys4::LacO(LEU2) his3::LacR-GFP(HIS3)*
4143	*MATa bar1Δ GAL-SMC4 trp1::LacO(TRP1, LEU2) lys4::LacO(LEU2) his3::LacR-GFP(HIS3)*
AVY461	*MATa bar1Δ trp1::LacO(TRP1, LEU2) lys4::LacO(LEU2) his3::LacR-GFP(HIS3)*
4424	*MATa bar1Δ SMC4-6HA::TRP1 rpn4::HygBR pdr5::NATR pGAL-SIC1-HA(URA3)*
AVY584	*MATa bar1Δ SMC4-6HA::TRP1 rpn4::HygBR pdr5::NATR*
4376	*MATa bar1Δ SMC4-6HA::TRP1 apc2::KANR apc2-4(TRP1) ura3::HIS3:GFP-TUB1(URA3)*
4366	*MATa bar1Δ SMC4-6HA::TRP1 GAL-pds1Δdb*
4370	*MATa bar1Δ SMC4-6HA::TRP1 GAL-pds1Δdb mad2::KANR*

### Cell cycle analysis

Overnight cultures were grown with extra adenine to decrease background green-fluorescence. Strains were grown at 30°C except for the temperature sensitive *apc2-4* mutant which was grown overnight at 24°C and at the non-permissive temperature during time-course experiments. The *GAL-SMC4-HA* strain was grown overnight in YPG to maintain *SMC4* expression. The *GAL-pds1Δdb* and *GAL-pds1Δdb mad2Δ* strains were grown overnight and synchronized in G1 in YPR to inhibit Pds1Δdb production followed by the addition of the galactose 10 minutes after the release. All other strains were grown at 30°C overnight in YPD. For synchrony, cells were diluted to OD 0.2 or 0.4 in YPD, YPG or YPR with mating pheromone (concentrations ranging from 1-2 μg/ml). After 2-2.5 hours, cells were monitored microscopically until G1 synchrony (85-100%) was reached. For the *apc2-4* mutant, cells were synchronized at 24°C for 2 hours followed by a half hour at 37°C. Mating pheromone was washed off with water and cells were released under experimental conditions. For arresting cells with nocodazole, the nocodazole (10 μg/ml as the final concentration) was added 10 minutes after G1 release. For inhibition of proteasome activity, MG132 (Sigma, 50 μg/ml as the final concentration) was added 40 minutes after the release.

### Biochemistry

Cell cultures were handled as described above except with no extra adenine addition. 10 ml of the culture at 0.4 OD were collected and pellets were kept at -80°C until protein was extracted. Pellets were resuspended in 1 ml 0.25M NaOH /1% 2-mercapotoethanol on ice for 10 minutes followed by the addition of 0.16 ml 50% TCA on ice for 10 minutes. Pellets were washed with 1 ml ice-cold acetone then resuspended in 2X sample buffer. Western blots used 1:1000 dilution of anti-HA (12CA5, Roche), a 1:10,000 dilution of anti-PSTAIRE (recognizing the PSTAIRE epitope of Cdk) or a 1:5000 dilution of HRP-conjugated anti-c-myc (Roche). Secondary antibody, HRP-conjugated goat anti-mouse (Pierce, Rockford, IL) was used at 1:5000. Western Lightning Chemiluminescence Reagent Plus (Perkin Elmer Life Sciences, Boston, MA) was used as per manufactures instructions.

### Microscopy (condensation assay)

The timing of chromosome condensation was monitored in living yeast using the previously developed LacO/GFP-LacI system to observe the *TRP1* and *LYS4* loci [15, 41]. The tandem repeated LacO sequences at the *TRP1* and *LYS4* loci on the right arm of chromosome IV are separated by 450 kb. The GFP dots are separated ∼1.06 μm in uncondensed chromosomes, whereas the two dots coalesce when chromosomes condense [15]. Yeast cells harboring this reporter system were divided into four categories based on cell morphology and chromosome structure (according to the GFP signal): (1) G1 cells with round cell morphology and two separated GFP dots, indicating uncondensed chromosomes; (2) S phase cells with small bud and two GFP dots; (3) G2/M cells, budded and a single GFP dot, which indicates condensed chromosomes; (4) Anaphase/Telophase cells with dumbbell shape and single or two GFP dots in each daughter cell following chromosome segregation. To determine the timing of chromosome condensation, the wild type cells harboring this LacO/GFP-LacI system were synchronized in G1 phase. After cells were released into the cell cycle, cells were collected every 10 minutes and subjected to observation by fluorescence microscopy. 100 randomly selected cells from each time point were scored into the corresponding category based on morphology and GFP signal as described above. Fluorescence and DIC microscopy with Alpha Plan Fluar 100x/1.45 objectives and a Zeiss Axio Plan II microscope were used for scoring and capturing images of live cells with a Zeiss Axiocam camera and AxioVision software.

## Abbreviations

Cdk: cyclin-dependent kinase
YPD: rich yeast growth medium with dextrose
YPG: rich yeast growth medium with galactose
YPR: rich yeast growth medium with raffinose
